# CD3 downregulation identifies high-avidity, multipotent SARS-CoV-2 vaccine– and recall antigen–specific Th cells with distinct metabolism

**DOI:** 10.1172/jci.insight.166833

**Published:** 2024-01-11

**Authors:** Arne Sattler, Stefanie Gamradt, Vanessa Proß, Linda Marie Laura Thole, An He, Eva Vanessa Schrezenmeier, Katharina Jechow, Stefan M. Gold, Sören Lukassen, Christian Conrad, Katja Kotsch

**Affiliations:** 1 Charité–Universitätsmedizin Berlin, corporate member of Freie Universität Berlin, Humboldt-Universität zu Berlin, and Berlin Institute of Health, Department for General and Visceral Surgery, Berlin, Germany.; 2 Charité–Universitätsmedizin Berlin, corporate member of Freie Universität Berlin, Humboldt-Universität zu Berlin, and Berlin Institute of Health, Department of Psychiatry and Neurosciences – Campus Benjamin Franklin, Berlin, Germany.; 3 Charité–Universitätsmedizin Berlin, corporate member of Freie Universität Berlin, Humboldt-Universität zu Berlin, and Berlin Institute of Health, Department of Psychosomatic Medicine – Campus Benjamin Franklin, Berlin, Germany.; 4 Charité–Universitätsmedizin Berlin, corporate member of Freie Universität Berlin, Humboldt-Universität zu Berlin, and Berlin Institute of Health, Department of Nephrology and Medical Intensive Care, Berlin, Germany.; 5 Charité–Universitätsmedizin Berlin, corporate member of Freie Universität Berlin, Humboldt-Universität zu Berlin, and Berlin Institute of Health, Center for Digital Health, Berlin, Germany.; 6 Universitätsklinikum Hamburg Eppendorf, Institut für Neuroimmunologie und Multiple Sklerose, Hamburg, Germany.

**Keywords:** Immunology, Vaccines, Cellular immune response, Organ transplantation, T cells

## Abstract

Functional avidity is supposed to critically shape the quality of immune responses, thereby influencing host protection against infectious agents including SARS-CoV-2. Here we show that after human SARS-CoV-2 vaccination, a large portion of high-avidity spike-specific CD4^+^ T cells lost CD3 expression after in vitro activation. The CD3^–^ subset was enriched for cytokine-positive cells, including elevated per-cell expression levels, and showed increased polyfunctionality. Assessment of key metabolic pathways by flow cytometry revealed that superior functionality was accompanied by a shift toward fatty acid synthesis at the expense of their oxidation, whereas glucose transport and glycolysis were similarly regulated in SARS-CoV-2–specific CD3^–^ and CD3^+^ subsets. As opposed to their CD3^+^ counterparts, frequencies of vaccine-specific CD3^–^ T cells positively correlated with both the size of the naive CD4^+^ T cell pool and vaccine-specific IgG levels. Moreover, their frequencies negatively correlated with advancing age and were impaired in patients under immunosuppressive therapy. Typical recall antigen–reactive T cells showed a comparable segregation into functionally and metabolically distinct CD3^+^ and CD3^–^ subsets but were quantitatively maintained upon aging, likely due to earlier recruitment in life. In summary, our data identify CD3^–^ T helper cells as correlates of high-quality immune responses that are impaired in at-risk populations.

## Abstract 2

<p>Activation-induced loss of CD3 marks high-avidity, multipotent SARS-Cov2 vaccine- and recall antigen-specific T cells with distinct metabolism that are reduced in the elderly and individuals with comorbidities.</p>

## Graphical abstract

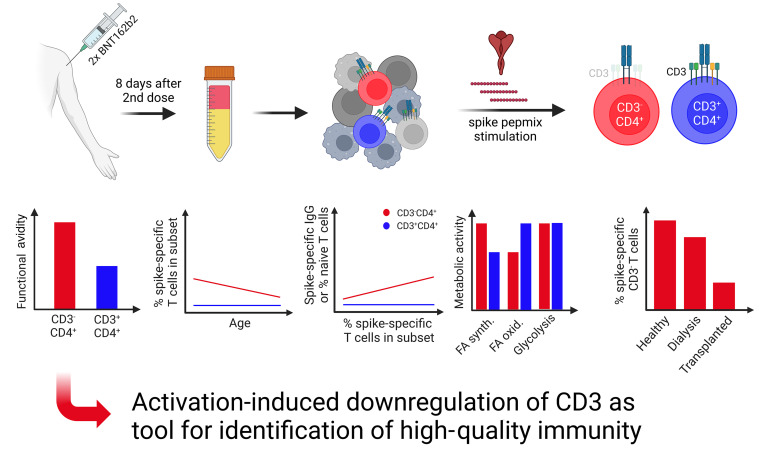

## Introduction

The quality of immune responses is considered to critically determine outcomes after infection, after vaccination, or in the context of antitumor immunity. However, only few tools enable assessment of parameters that unequivocally reflect functional superiority of molecular or cellular immune features in humans. Among those, quantification of neutralizing antibody levels is key to estimate antiviral protection, e.g., against SARS-CoV-2 (1) or influenza virus (2). Long-term nonprogression after human immunodeficiency virus (HIV) infection has been found to correlate with increased quantities of polyfunctional T cells (3, 4) that are also characteristically enriched in individuals experiencing mild versus severe SARS-CoV-2 infections (5). Vice versa, immunophenotyping of patients with COVID-19 suggested that preconditions such as higher comorbidity index or advanced age are associated with reduced frequencies of virus-reactive IFN-γ–secreting T helper (Th) cells in infected individuals (6), contributing to undesirable COVID-19 prognosis. The concept of altered T cell function in severe disease was further substantiated by Bacher et al., showing that SARS-CoV-2–specific, in vitro–generated short-term T cell lines of nonhospitalized patients responded to lower antigen concentrations than those from severe cases (7), indicating higher functional avidity.

The limitations of such a labor- and time-intensive approach for large-scale individual evaluation have been recently overcome by data from Loyal et al., revealing that CD4^+^ T cells with reduced surface CD3 expression show increased functional avidity in SARS-CoV-2–infected individuals (8). Although loss of TCR (9) or CD3 expression in response to T cell stimulation has been described earlier for both CD4^+^ (10) and CD8^+^ subsets (11), CD3-diminished or -negative T cells have been excluded from studies comprehensively assessing vaccine-specific immunity so far. We therefore set out to compare classical CD3^+^CD4^+^ Th cell responses with those of their CD3^–^ counterparts in healthy individuals after standard SARS-CoV-2 mRNA vaccination and in response to viral recall antigens. Our analyses demonstrate that vaccine-specific CD3^–^ Th cells are superior with respect to their functional avidity and show distinct metabolic programming, and their reduced mobilization into novel vaccine-induced immune responses is characteristic for an aging immune system. In summary, our data suggest that identification of high-avidity Th cells based on activation-induced downregulation of CD3 could serve as an important tool for risk assessment of vulnerable patient groups in the context of vaccination or infection and might facilitate selection of superior clones for therapeutic purposes such as adoptive T cell transfer.

## Results

### Segregation of SARS-CoV-2 spike protein–specific CD4^+^ T cells into CD3^+^ and CD3^–^ subsets after mRNA vaccination.

**Figure 1:**
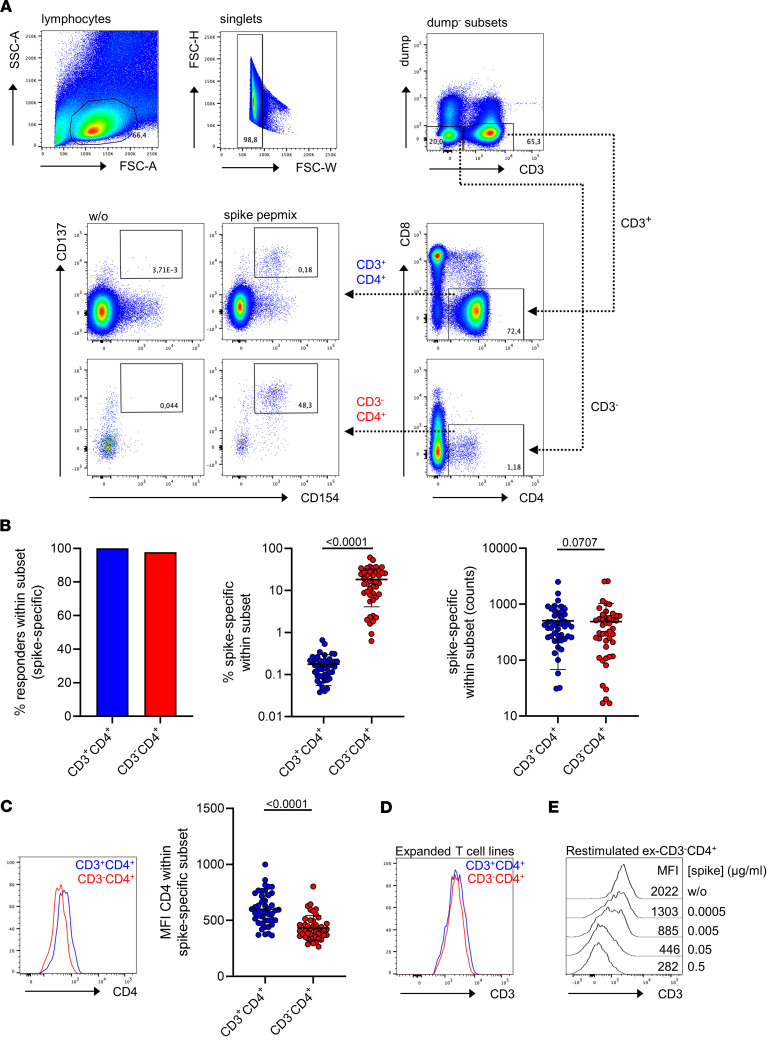
Identification and quantification of vaccine-reactive CD3^–^CD4^+^ Th cells. (**A**) PBMCs were stimulated or not with spike peptide mix for 16 hours. Specific CD4^+^ Th cells were identified within the CD3^+^ and CD3^–^ compartments according to coexpression of CD154 and CD137. (**B**) Portions of responders (left), frequencies (middle, *n* = 44), and counts (right, *n* = 44) of specific cells in both compartments. (**C**) CD4 expression levels in the indicated subsets as reflected by mean fluorescence intensity (MFI). *n* = 44. Statistical analyses in **A**–**C** were performed using paired, 2-tailed Wilcoxon test throughout. Graphs show means ± SD. (**D**) CD3 expression levels of specific FACS-purified, CD3^–^CD4^+^- or CD3^+^CD4^+^ -derived T cell lines after 10 days of in vitro expansion, representative for *n* = 3. (**E**) CD3 expression analysis of in vitro–expanded, CD3^–^CD4^+^-derived T cell lines after specific restimulation with titrated amounts of antigen, representative for *n* = 3. SSC, side scatter; FSC, forward scatter.

For detection of SARS-CoV-2 spike-specific CD4^+^ Th cells, PBMCs were obtained 8 ± 1 days after the second dose of a 2-dose standard mRNA vaccination (BioNTech/Pfizer) of virus-naive individuals and stimulated with an overlapping peptide pool encompassing the complete viral spike protein. After gating on dump^–^ live lymphocytes (for details, see Methods section), specific cells were assessed within the CD3^+^ and CD3^–^ compartments. Interestingly, pre-gating on CD3^–^ T cells revealed a small CD4-positive to -dim subset enriched for specific CD154^+^CD137^+^ T cells (Figure 1A). Overall, spike-specific T cells were identified in all but 1 donor in both compartments (Figure 1B, left). Relative frequencies within the CD3^–^CD4^+^ subset were significantly enriched around 10-fold (mean: 17.92% ± 2.120%) as compared with the CD3^+^CD4^+^ fraction (mean: 0.1759% ± 0.01807%) (Figure 1B, middle). Cell counts within both subpopulations did not exhibit significant differences but tended to be moderately lower within the CD3^–^CD4^+^ compartment (Figure 1B, right) that was further characterized by significantly reduced CD4 expression (Figure 1C) as reflected by lower mean fluorescence intensity (MFI) levels. Quantification of spike-specific CD3^+^CD4^+^ and CD3^–^CD4^+^ T cells with 4 commercially available anti-CD3 clones conjugated to the same fluorochrome (Supplemental Figure 1A; supplemental material available online with this article; https://doi.org/10.1172/jci.insight.166833DS1) yielded comparable results (Supplemental Figure 1B). Both subsets were largely CD28^+^ and did not show expression of the NK cell–associated molecules CD16, CD56, and CD94 (Supplemental Figure 2), suggesting that they are bona fide T cells. To address whether these vaccine-specific T cells lacked CD3 expression ex vivo or downregulated the molecule in a stimulation-dependent manner, PBMCs were stained and analyzed as before, with the exception that CD3 was labeled either before or after stimulation. In pre-experiments, we determined CD3 prestaining to be stable over the culture period with some acceptable loss of fluorescence intensity (Supplemental Figure 3A, upper left vs. lower left plot). Of note, only few spike-specific T cells were detectable within CD3^–^ T cells when samples were stained prior to stimulation. In contrast, more than 15-fold higher frequencies of antigen-reactive CD154^+^CD137^+^ T cells were identified when samples were labeled after stimulation (Supplemental Figure 3, A and B), indicating that CD3 is largely downregulated as a consequence of stimulation in a subset of specific T cells. To substantiate this notion, spike-specific CD3^–^CD4^+^CD154^+^CD137^+^ T cells were MACS–pre-enriched, followed by FACS purification (Supplemental Figure 3C), and expanded for 10 days in vitro with cytokines and autologous feeder cells in the absence of antigen. CD3 (re-) expression levels in CD3^–^CD4^+^ and CD3^+^CD4^+^ -derived T cell lines were comparable after the expansion period (Figure 1D). Upon antigen-specific restimulation, CD3^–^CD4^+^-derived lines downregulated CD3 again in an antigen concentration-dependent manner as reflected by decreasing MFI levels (Figure 1E). We next assessed whether the spike-specific CD3^–^CD4^+^ population could also be detected after single-peptide stimulation. Several reports (8, 12, 13) suggested that a 15-mer encompassing amino acids (aa) 816–830 is recognized by 20%–60% of healthy probands and up to 100% in individuals expressing the MHC class II allele DPA1*01:03 (8). We therefore stimulated PBMCs of a vaccinated, DPA1*01:03^+^ heterozygous blood donor with full-spike peptide mix versus aa 816–830 peptide. As already demonstrated, full-spike stimulation yielded similar counts of specific cells in both compartments, associated with an enrichment of IFN-γ^+^ cells in the CD3^–^CD4^+^ subset. After aa 816–830 stimulation, specific cells were detectable in both compartments with a quantitative dominance of CD3^+^CD4^+^ over CD3^–^CD4^+^ T cells. Vice versa, frequencies of IFN-γ producers were elevated in the CD3^–^CD4^+^ subpopulation (Supplemental Figure 4).

### Correlation of spike-reactive CD3^–^CD4^+^ T cells with specific IgG levels, age, and the size of the naive T cell pool.

**Figure 2:**
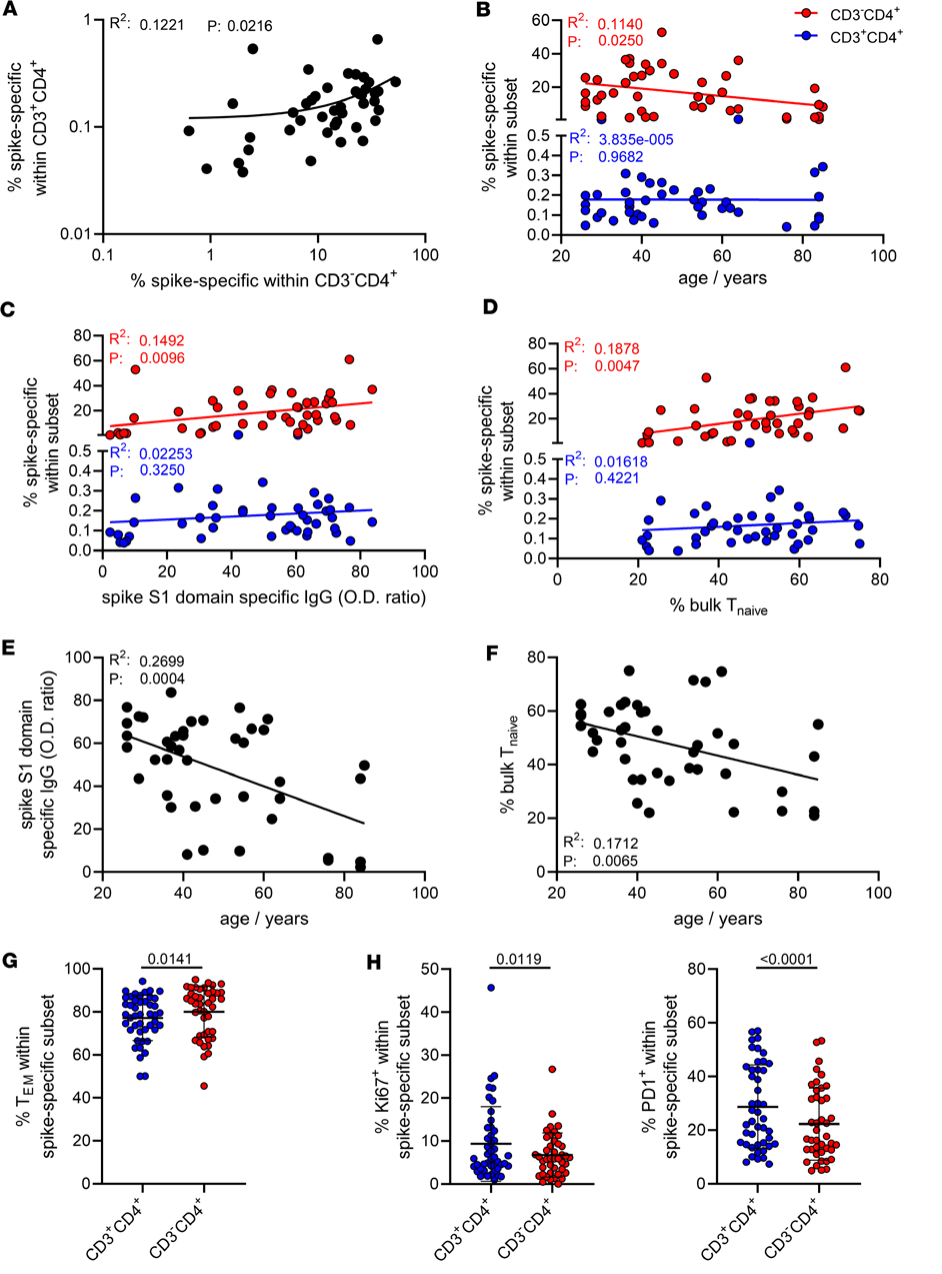
Vaccine-specific CD3^–^CD4^+^ Th cells decline with age and positively correlate with specific antibody levels and frequencies of naive T cells. Frequencies of spike-specific CD3^–^CD4^+^ and CD3^+^CD4^+^ Th cells were determined as before and correlated (**A**) with each other (*n* = 44), (**B**) proband age (*n* = 44), (**C**) spike S1 domain–specific IgG levels (*n* = 44), or (**D**) frequencies of bulk naive CD45RO^–^CD62L^+^ Th cells (*n* = 42). Further analyses address the interrelation of age with specific antibody levels (**E**) or (**F**) frequencies of bulk naive CD45RO^–^CD62L^+^ Th cells (both *n* = 42). (**G**) Portions of CD45RO^+^CD62L^–^ effector/memory Th cells in both subsets. (**H**) Quantified frequencies of spike-specific proliferating Ki67^+^ (left) or in vivo–activated PD-1^+^ (right) Th cells (both *n* = 44); statistical analyses were performed using simple linear regression (**A**–**F**) or paired, 2-tailed Wilcoxon test (**G** and **H**). Graphs show means ± SD. PD-1, programmed cell death 1.

We detected a moderately positive correlation between specific T cell frequencies in both subsets (Figure 2A). Over 7 decades of life, relative portions of vaccine-specific CD3^–^CD4^+^ T cells slightly but significantly decreased, whereas frequencies of their CD3^+^ counterparts remained constant (Figure 2B). Importantly, only frequencies of spike-specific CD3^–^CD4^+^ T cells, but not of CD3^+^CD4^+^, positively correlated with spike S1–specific IgG levels (Figure 2C). Furthermore, frequencies of the CD3^–^CD4^+^ fraction were strongly associated with increased portions of bulk naive CD3^+^CD4^+^CD45RA^+^CD62L^+^ T cells (Figure 2D; with the gating strategy depicted in Supplemental Figure 5A). Advanced age was strongly associated with both reduced antibody levels (Figure 2E) as well as frequencies of bulk naive T cells (Figure 2F). In a limited number of individuals, we analyzed frequencies of specific T cells at different time points (range: 19–262 days, mean: 98.65 days) after vaccination in a cross-sectional manner. Specific CD3^+^CD4^+^ and CD3^–^CD4^+^ subsets were detectable in all samples and remained comparably stable over the observation period with no significant decline in frequencies (Supplemental Figure 6). Vaccine-specific CD3^–^CD4^+^ T cells showed slightly, but significantly, higher frequencies of CD45RO^+^CD62L^–^ effector/memory T cells (Figure 2G, gating strategy in Supplemental Figure 5A); interestingly, they were characterized by reduced portions of proliferating Ki67^+^ cells (Figure 2H, left, with gating strategy in Supplemental Figure 5A), in line with diminished percentages of recently in vivo–activated PD-1^+^ cells (Figure 2H, right, with gating strategy in Supplemental Figure 5A). Females showed higher frequencies of spike-specific CD3^–^CD4^+^ but not CD3^+^CD4^+^T cells than males (Supplemental Figure 7A); both groups were characterized by a comparable age composition (Table 1).

In summary, CD3^–^CD4^+^ frequencies associate with vaccine-specific titers and are diminished with increasing age, and their quantitative recruitment into SARS-CoV-2–specific vaccine responses is augmented in individuals harboring elevated portions of naive T cells.

### Distinct metabolic features characterize vaccine-specific CD3^–^CD4^+^ T cells.

**Figure 3:**
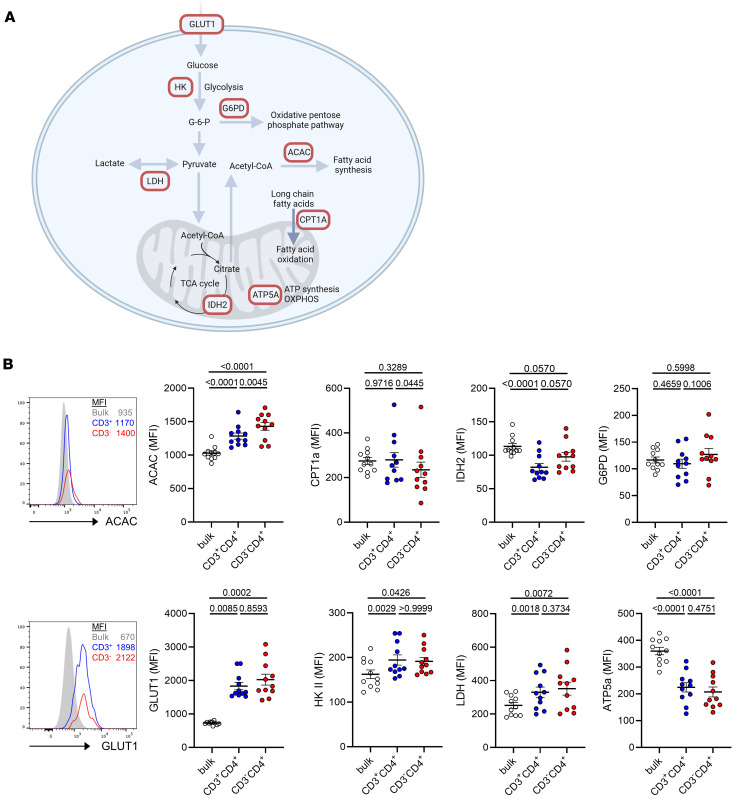
Single-cell metabolic pathway analysis in spike-specific CD3^–^CD4^+^ T cells. (**A**) Overview of cell metabolism and key metabolic proteins targeted by flow cytometry. (**B**) PBMCs were stimulated or not with spike peptide mix for 16 hours. The expression level (MFI) of key metabolic proteins was analyzed in spike-specific (CD154^+^CD137^+^) CD3^+^CD4^+^ versus CD3^–^CD4^+^ T cells versus nonspecific CD154^–^CD137^–^CD3^+^CD4^+^ “bulk” control T cells within the same sample. Histograms (upper and lower left) show exemplary expression characteristics in the 3 subsets for ACAC and GLUT1. *n* = 11; statistical analyses were performed using paired 1-way ANOVA (ACAC, CPT1a, G6PD, ATP5a, LDH) or Friedman test (IDH2, GLUT1, HK2). Where applicable, graphs show means ± SD.

Recent reports have defined key metabolic proteins, including rate-limiting enzymes, for robust single-cell assessment of the metabolic state by flow or mass cytometry, using validated antibodies (14, 15). For pioneering quantification of multimodal metabolic features within rare antigen-specific T cells, we designed a FACS panel characterizing components critical for glucose uptake (glucose transporter 1, GLUT1), glycolysis (hexokinase 2, HK2), tricarboxylic acid cycle (isocitrate dehydrogenase 2, IDH2), oxidative pentose phosphate pathway (glucose-6-phosphate dehydrogenase, G6PD), ATP synthesis (ATP synthase subunit α, ATP5A), lactate metabolism/aerobic glycolysis (lactate dehydrogenase, LDH), and fatty acid synthesis (acetyl-CoA carboxylase α, ACAC) and oxidation (carnitine palmitoyl-transferase 1A, CPT1A). An overview of the covered metabolic pathways is depicted in Figure 3A. To assess immune metabolism in specific T cells, PBMCs were stimulated and spike-reactive CD154^+^CD137^+^ CD3^+^ and CD3^–^ Th cell subsets identified as before, along with expression analysis of metabolic markers based on their individual MFI. In all analyses, the nonreactive CD3^+^CD4^+^CD154^–^CD137^–^ T cell population (termed “bulk” in the respective figures) served as internal control. Overall, specific CD3^+^ and CD3^–^ T cell subsets similarly employed glycolysis after activation, as mirrored by comparable upregulation of GLUT1 and HK2 over controls; the same applied to expression levels of LDH, a critical enzyme during aerobic glycolysis, which is a hallmark of metabolic reprogramming in activated T cells. Interestingly, ATP synthesis as reflected by ATP5A expression was substantially downregulated in both subsets as compared with nonreactive controls. Importantly, the fatty acid synthesis pathway represented by ACAC expression was significantly upregulated in CD3^–^ over CD3^+^ T cells and controls. Concomitantly, specific CD3^–^ but not CD3^+^ Th cells reduced fatty acid oxidation, given their significantly lower expression levels of CPT1A (Figure 3B). Spike-specific CD3^–^ Th cells were further characterized by reduced activation-dependent downregulation of IDH2 over their CD3^+^ counterparts and a moderate increase of G6PD expression over both CD3^+^ and control Th cells (Figure 3B).

As a conclusion, spike-specific CD3^–^CD4^+^ T cells stand out with respect to their augmented metabolic switch to fatty acid synthesis, being associated with significant downregulation of fatty acid oxidation as compared with CD3^+^CD4^+^ and control Th cells.

### CD3^–^CD4^+^ T cells are functionally superior to their CD3^+^ counterparts.

**Figure 4:**
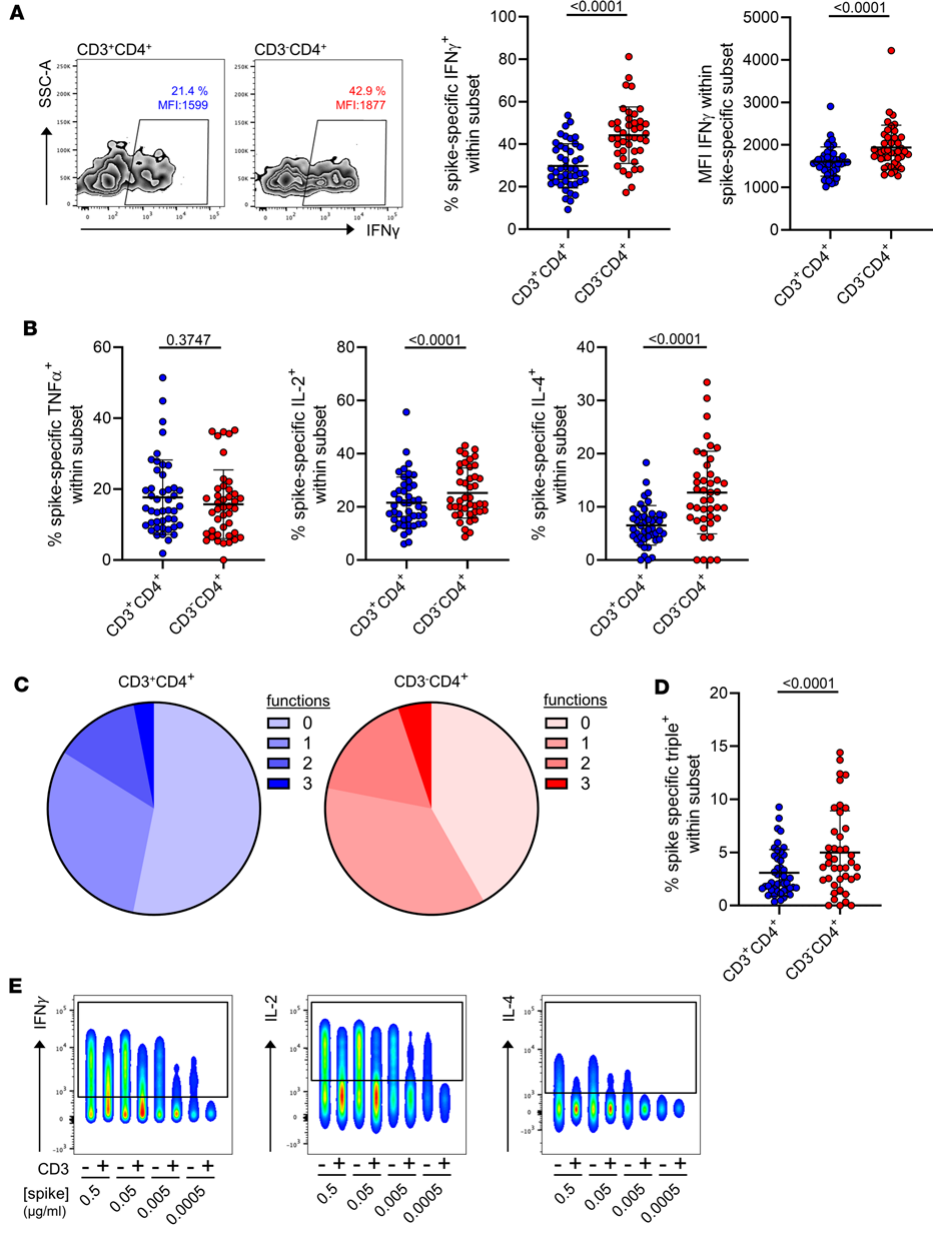
Differential cytokine production capacity by spike-specific CD3^–^CD4^+^ and CD3^+^CD4^+^ Th cell subsets. PBMCs were stimulated or not with spike peptide mix for 16 hours. Specific CD4^+^ Th cells were identified as before and further assessed intracellularly for cytokine production. (**A**) Exemplary zebra plots (left) and summary of frequencies (middle, *n* = 44) or per-cell expression levels (right, *n* = 44) of IFN-γ^+^ T cells within both spike-specific populations. (**B**) Frequencies TNF-α (left), IL-2 (middle), or IL-4 (right) expressing spike-specific T cells, all *n* = 44. (**C**) Mean frequencies of specific cells showing 0, 1, 2, or 3 functions with respect to IFN-γ–, TNF-α–, and IL-2 expression in both subsets and (**D**) statistics for IFN-γ^+^TNF-α^+^IL-2^+^ polyfunctional T cells. *n* = 43. Statistical analysis with paired, 2-tailed *t* test (**A**, left; **B**, right) or paired, 2-tailed Wilcoxon test (all other graphs). Where applicable, graphs show means ± SD. (**E**) Production of IFN-γ, IL-2, and IL-4 in in vitro–expanded, FACS-sorted spike-specific CD3^–^CD4^+^ versus CD3^+^CD4^+^ T cell subsets after restimulation with titrated concentrations of antigen, representative for *n* = 2 donors out of independent experiments.

To assess the functional potential of spike-specific CD3^–^CD4^+^ vs. CD3^+^CD4^+^ T cells, cytokine production was analyzed on the population level and per cell with the gating strategy depicted in Supplemental Figure 5B. The CD3^–^CD4^+^ subset contained significantly higher frequencies of IFN-γ^+^, IL-2^+^, and IL-4^+^ but not TNF-α^+^ cells (Figure 4, A and B). This difference also applied to the amount of IFN-γ (Figure 4A, right) and IL-4 (Supplemental Figure 7B, right) expressed per cell as evidenced by higher MFI levels and to frequencies of IFN-γ^+^TNF-α^+^IL-2^+^ coproducing polyfunctional T cells (Figure 4, C and D). Interestingly, MFI levels of the growth factor IL-2 were significantly reduced in the CD3^–^ subset (Supplemental Figure 7B, middle). To validate data that functional superiority relies on higher functional avidity (8), PBMCs were stimulated with spike peptide mix; CD154^+^ cells were pre-enriched by MACS, followed by FACS purification of specific CD3^–^CD4^+^ and CD3^+^CD4^+^ subpopulations (Supplemental Figure 3C) and in vitro expansion. After specific restimulation with titrated amounts of antigen, the CD3^–^CD4^+^ subset contained substantially higher frequencies of cells producing IFN-γ, IL-2, or IL-4 than their CD3^+^ counterparts regardless of what antigen concentration was used: the CD3^–^CD4^+^ population still produced cytokines at low antigen concentrations, at which the CD3^+^CD4^+^ population was already cytokine negative (Figure 4E).

### Quantitative, functional, and metabolic features of typical recall antigen–specific CD3^–^CD4^+^ T cells.

**Figure 5:**
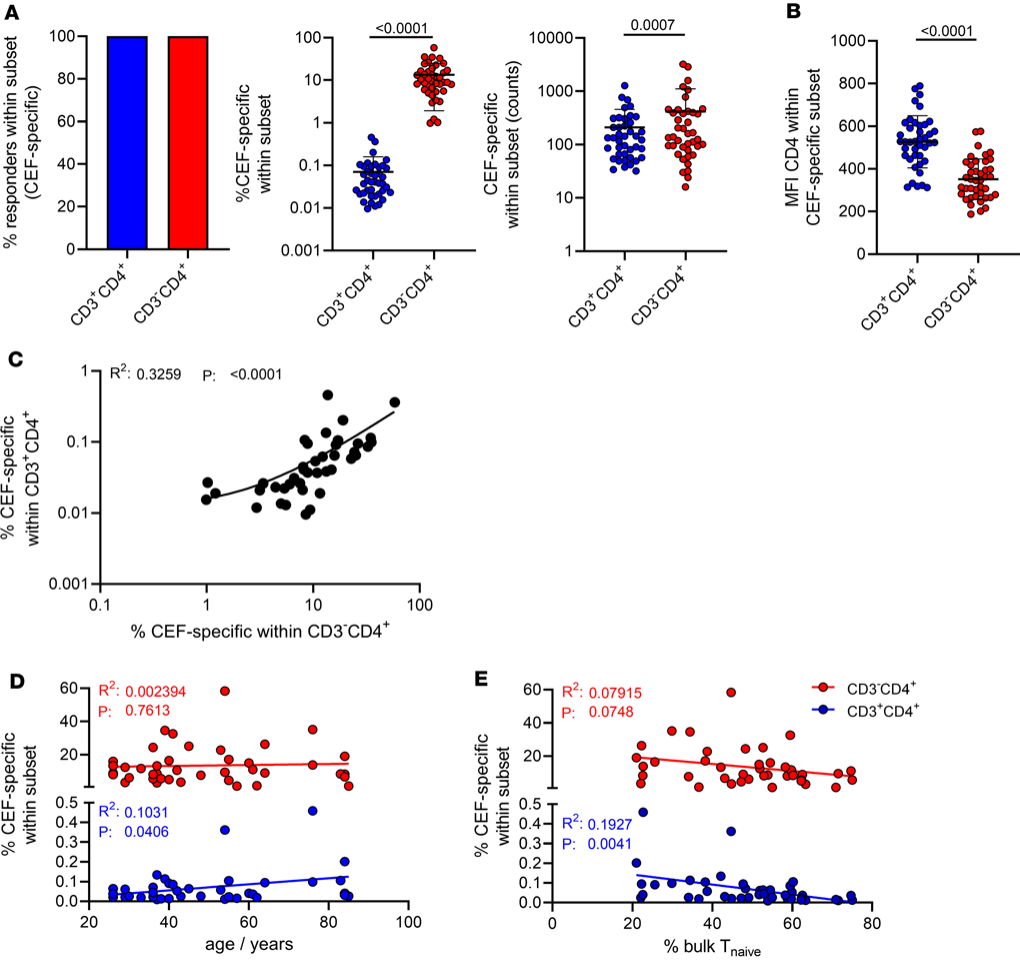
Characterization of the CEF-specific CD3^–^CD4^+^ T cell response. PBMCs were stimulated or not with CEF peptide mix for 16 hours. Specific CD4^+^ Th cells were identified within the CD3^+^ and CD3^–^ compartments according to coexpression of CD154 and CD137. (**A**) Portions of responders (left), frequencies (middle), and counts (right) of specific cells in both compartments. (**B**) CD4 expression levels in the indicated subsets as reflected by MFI. (**C**–**E**) **C** depicts the correlation between frequencies of both subsets, whereas **D** and **E** show correlations between frequencies and age or the percentage of bulk naive CD45RO^–^CD62L^+^ Th cells, respectively. *n* = 41 throughout; statistical analyses were performed using paired, 2-tailed Wilcoxon test (**A**); paired, 2-tailed *t* test (**B**); or simple linear regression (**C**–**E**). Where applicable, graphs show means ± SD.

We next addressed whether separation into CD3^–^CD4^+^ and CD3^+^CD4^+^ T cell subsets could not only be detected in de novo–vaccinated, previously virus-naive individuals but also account for typical recall antigen–specific responses. Therefore, analyses were conducted as before for Th cells reactive to a peptide mix consisting of CMV, EBV, and influenza antigens, termed CEF, that were detectable in all individuals (Figure 5A, left). In analogy to their spike-specific counterparts, CEF-specific T cells also segregated into CD3^+^CD4^+^ and CD3^–^CD4^+^ subsets, with the latter bearing significantly higher frequencies (mean: 0.07% ± 0.01385% vs. 13.39% ± 1.794%) (Figure 5A, middle), showing significantly higher counts (Figure 5A, right) and a strong downregulation of CD4 (Figure 5B). In addition, frequencies of both subpopulations showed a highly significant positive correlation (Figure 5C). Of note, as opposed to our findings for spike-specific T cells, portions of CD3^–^CD4^+^ CEF-specific T cells did not diminish with age, whereas frequencies of the CD3^+^CD4^+^ subset increased slightly, but significantly, with advanced age (Figure 5D). Both subsets showed a negative correlation with frequencies of naive T cells; however, this observation reached significance only for the CD3^+^CD4^+^ population (Figure 5E).

**Figure 6:**
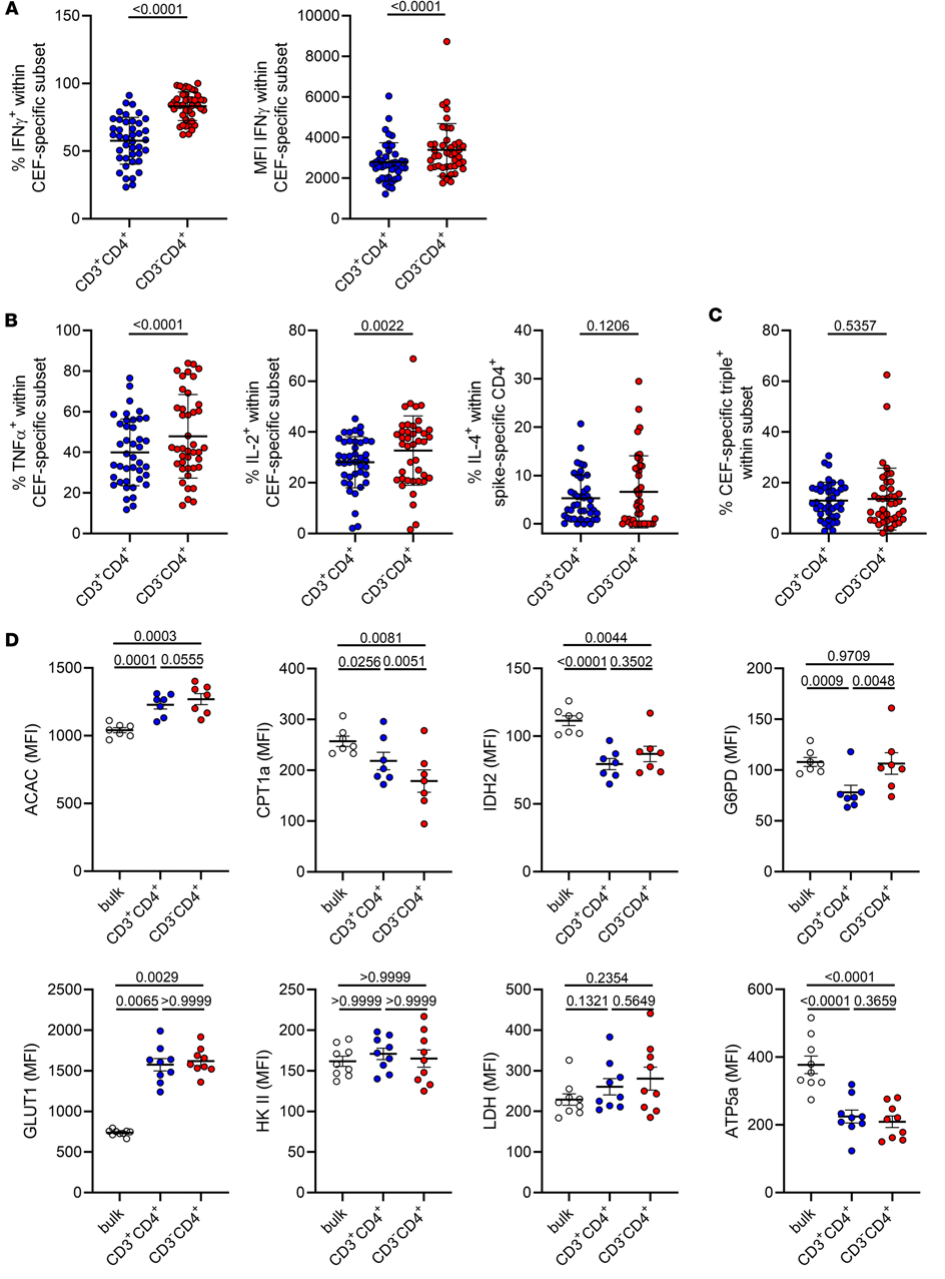
Functional and metabolic assessment of CEF-specific CD3^–^CD4^+^ and CD3^+^CD4^+^ Th cell subsets. PBMCs were stimulated or not with CEF peptide mix for 16 hours. Specific CD4^+^ Th cells were identified as before and further assessed intracellularly for cytokine production. (**A**) Frequencies (left, *n* = 42) and expression levels (right, *n* = 42) of IFN-γ^+^ T cell subsets specific for CEF. (**B**) Frequencies of TNF-α– (left), IL-2– (middle), or IL-4– (right) expressing CEF-specific T cells and (**C**) those of IFN-γ–, TNF-α–, and IL-2– coexpressing polyfunctional T cells. *n* = 41 for all analyses. Statistical analysis with paired, 2-tailed *t* test (**A**, left, **B**, middle) or paired, 2-tailed Wilcoxon test (remaining graphs). (**D**) Expression levels (according to MFI) of the indicated key metabolic proteins were analyzed in CEF-specific (CD154^+^CD137^+^) CD3^+^CD4^+^ versus CD3^–^CD4^+^ T cells versus nonspecific CD154^–^CD137^–^CD3^+^CD4^+^ bulk control T cells within the same sample. *n* = 7 (upper panels) and 9 (lower panels); statistical analyses were performed using paired 1-way ANOVA (ACAC, CPT1a, IDH2, G6PD, LDH, ATP5a) or Friedman test (GLUT1, HK2). Where applicable, graphs show means ± SD.

Significantly elevated frequencies of cytokine-positive cells were also detected within CEF-reactive CD3^–^CD4^+^ as compared with CD3^+^CD4^+^ T cells; this observation was most pronounced for IFN-γ, and applied to TNF-α and IL-2, but excluded IL-4 or polyfunctionality (Figure 6, A–C). Significantly higher per-cell expression levels within the CD3^–^CD4^+^ compartment were only noted for IFN-γ (Figure 6A, right), whereas a slight decrease was observed for IL-2 (Supplemental Figure 7C).

Interestingly, CEF-specific Th cells revealed similar metabolic features as already determined for the spike-specific population. Thus, fatty acid synthesis as reflected by ACAC expression levels was particularly upregulated in the CEF-specific CD3^–^CD4^+^ subset, along with significantly diminished fatty acid oxidation mirrored by reduced CPT1a expression and reduced downregulation of G6PD as compared with the CD3^+^CD4^+^ subset. Although HK2 levels were only slightly upregulated in both CEF subpopulations over controls, significantly elevated expression of GLUT1 over controls is suggestive of increased glucose metabolism (Figure 6D).

### Quantitative and functional impairment of spike-specific CD3^–^CD4^+^ T cells in patients under immunosuppressive therapy.

**Figure 7:**
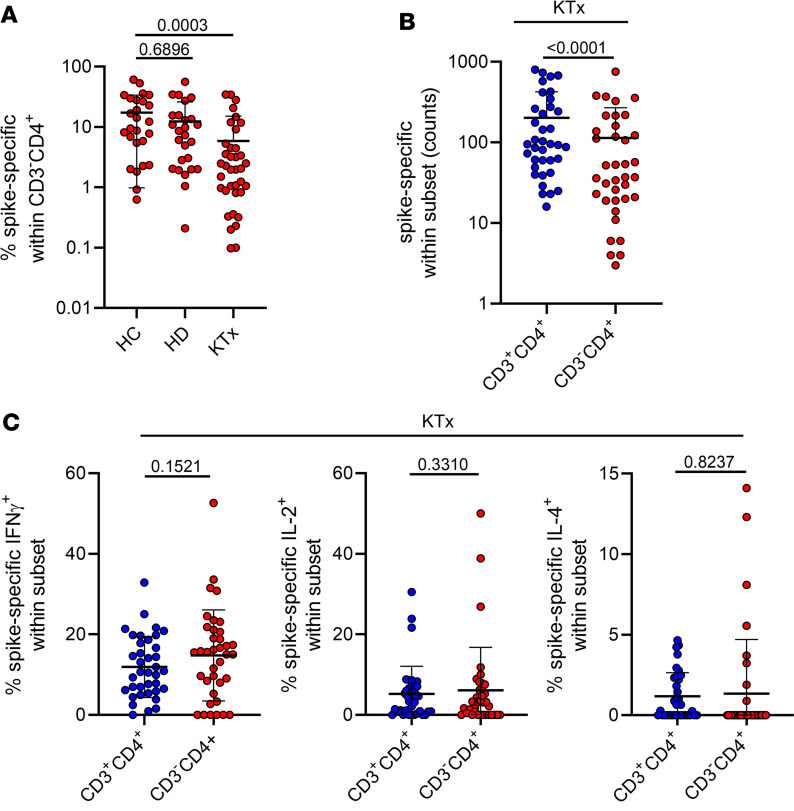
Vaccine-specific CD3^–^CD4^+^ Th cells in patients on dialysis and kidney transplant recipients. (**A**) Spike-specific CD3^–^CD4^+^ T cells were identified as before; frequencies were quantified in age-matched healthy controls (HC), patients on hemodialysis (HD), and kidney transplant recipients under immunosuppressive treatment (KTx). Frequencies of (**B**) spike-specific CD3^–^CD4^+^ and CD3^+^CD4^+^ Th cell subsets and (**C**) portions of IFN-γ– (left), IL-2– (middle), and IL-4– (right) expressing Th cell subsets in kidney transplant recipients. *n* = 26 (HC)/26 (HD)/37 (KTx). Statistical analysis was performed with Kruskal-Wallis-test (**A**); paired, 2-tailed *t* test (**B**, left); or paired, 2-tailed Wilcoxon test (all other graphs). Where applicable, graphs show means ± SD.

Patients with comorbidities, including those on dialysis or after solid organ transplantation, are prone to experience more severe COVID-19 disease courses (16, 17) and show multiple impairments in humoral and cellular SARS-CoV-2 vaccine–specific immunity (18, 19). We therefore examined vaccination-induced CD3^–^CD4^+^ T cells in patients with renal insufficiency on dialysis and kidney transplant recipients under standard immunosuppressive therapy. Both groups were age-matched with healthy probands (Table 2); all samples were consistently examined 8 ± 1 days after the second standard mRNA vaccination. Interestingly, in line with previous reports on conventional Th cell responses (18, 20, 21), patients on dialysis were characterized by only moderate reduction of spike-specific CD3^–^CD4^+^ T cells (mean: 12.36% ± 2.671%) in comparison with controls (17.31% ± 3.202%). However, transplant recipients showed a significant reduction of CD3^–^CD4^+^ T cell frequencies (mean: 5.871% ± 1.513%) compared with healthy probands (Figure 7A) and a significant expansion of specific CD3^+^CD4^+^ counts at the expense of the CD3^–^CD4^+^ population (Figure 7B). Of note, the CD3^–^ subset in transplant recipients did not exhibit characteristics of superior cytokine production (Figure 7C) that were observed in healthy individuals (Figure 4, A–D). In summary, spike-specific CD3^–^CD4^+^ T cells of transplant recipients under immunosuppressive medication are numerically and functionally devoid of typical hallmarks found in healthy probands.

## Discussion

Definition and straightforward analysis of key parameters reflecting the quality of vaccine-specific immunity are of particular interest for predicting disease outcomes in at-risk groups. Here we used differential CD3 expression on human Th cells as an easily applicable tool that may explain fundamental aspects of unsatisfactory immune responses after vaccination of healthy older individuals or patients with preconditions. At the same time, it could be used as a new biomarker for general assessment of high-quality immune responses. Our data on SARS-CoV-2 mRNA vaccine–specific Th cells build on the recent finding of a novel CD4^+^ subpopulation that rapidly downregulates CD3 expression in response to in vitro stimulation in SARS-CoV-2–infected individuals (8). After SARS-CoV-2 mRNA vaccination, we found this population to be of comparable size as its “classical” CD3^+^ counterpart as reflected by similar total counts. With respect to typical viral recall antigen–specific responses, the CEF-specific CD3^–^ subset even outnumbered conventional CD3^+^ Th cells by a factor of 2, highlighting that a substantial fraction of the specific Th cell pool had been excluded in commonly employed FACS-dependent stimulation assays so far. Based on our data, inclusion of the CD3^–^ subset for studying vaccine-specific cellular immunity seems of prime importance given their superior functional avidity as reflected by a lower activation threshold and increased cytokine secretion and polyfunctionality on a population- and per-cell level. Key characteristics of vaccine-reactive CD3^–^ T cells further encompass distinct regulation of fatty acid metabolism, their positive correlation with the size of the naive T cell pool, and an age-dependent decline that was not observed for conventional CD3^+^ Th cells.

With the advent of SARS-CoV-2, comprehensive analysis of antiviral immunity in large patient cohorts provided new insights into how cellular immunity measures might correlate with disease outcomes. So far, however, only few studies exist that bridge individual predisposing factors with immune measures beyond antibody levels that are distinctly regulated in at-risk groups, leading to undesired outcomes after infection or vaccination. In that respect, we previously showed that advanced age or increased comorbidity index shape the specific Th cell response toward reduced IFN-γ production in SARS-CoV-2–infected patients (6), a feature that was recapitulated in aged SARS-CoV-2 mRNA vaccinees (22). In the light of our data and in concert with findings on impaired functional avidity of Th cell responses in severe COVID-19 (7), the aforementioned functional limitations are likely resulting from reduced recruitment of high-avidity T cells with superior cytokine production capacity. Whereas a relation between advanced age and reduced thymic output (leading to up to 50% loss of naive T cell counts in individuals aged >60 years as compared with adolescents) is long anticipated to impair immunocompetence in older people (23), our study suggests that in particular high-avidity responses are quantitatively compromised, resulting from an increasingly limited naive T cell pool. In support of this finding, bulk naive T cell counts prevaccination proved to be the best predictor for protective titers after A(H1N1)pdm09 influenza vaccination, with both measures being diminished with advancing age (24).

Our comprehensive characterization of specific CD3^–^ T cell biology included assessment of cell metabolism. Focusing on validated antibodies detecting key protein targets, mostly rate-limiting enzymes (14, 15, 25), this innovative cytometry-based approach allows a sensitive quantification of metabolic activity with single-cell resolution, thereby enabling us to apply this technology to the analysis of rare antigen-specific T cells. In general, protein-based analyses might more accurately reflect cells’ metabolic state than transcriptome-based studies, given that regulation of metabolic pathways has been shown to dominantly occur on the translational rather than transcriptional level (26). As a key finding, our data highlight significant differences in fatty acid metabolism between antigen-specific CD3^–^ and CD3^+^ subsets. The fact that ACAC expression was highest in the CD3^–^ subset suggests increased fatty acid demand being characteristic for this subset upon activation, along with reduced fatty acid oxidation via CPT1A, possibly resulting from its blockade by ACAC synthesis product malonyl-CoA (27). Of note, mice with T cell–specific ACAC deletion fail to generate efficient Th1 responses, produce less IFN-γ, and succumb early to *M*. *tuberculosis* infection (28). Together with the finding that ACAC deletion or blockade reduces Th1-mediated inflammation in experimental colitis (29) and limits excessive IFN-γ production in human autoimmunity (30), these aspects contextualize the increased demand for fatty acid synthesis with superior execution of effector functions, including IFN-γ production, in spike- and CEF-specific CD3^–^ T cells.

Our findings of increased GLUT1, HK2, and LDH expression in both T cell subsets verify several other studies on bulk T cells demonstrating augmented aerobic glycolysis (also known as the Warburg effect) upon polyclonal activation (31, 32). At the same time, as opposed to the bulk analyses performed by Ahl at al. using αCD3/CD28 stimulation (14), our results highlight that not all metabolic pathways are comparably upregulated upon antigen-specific (i.e., physiologic) TCR triggering. This applied to IDH2 and ATP5A, showing reduced expression over unstimulated controls in spike-activated T cells, which is concomitant with the well-described switch from oxidative metabolism to aerobic glycolysis to support rapid effector function after activation (33, 34).

Of note, metabolic protein expression patterns similarly applied to spike- and CEF-specific responses, arguing for common metabolic requirements in established memory/effector Th cells regardless of their specificity.

As another common motif for spike- and CEF-specific responses, we found CD3^–^CD4^+^ T cells to express less IL-2 on a per-cell basis. The fact that the quantity of autocrine IL-2 could directly impact specific T cell expansion (35) might explain why we detected slightly, but significantly, reduced frequencies of proliferating and in vivo–activated PD-1^+^ T cells within this subset (Figure 2H). Interestingly, this aspect seems counterintuitive, based on the assumption that high-avidity T cells exhibit superior proliferation capacity. However, this phenomenon might be explained by the comparably high antigen dose and sustained local protein availability associated with mRNA vaccination (36, 37). In that context, the functional potential of high-avidity cytotoxic T cells was demonstrated to be critically linked to antigen dose, with supraoptimal concentrations limiting cellular expansion in vivo and in vitro (38).

As a side note, we could also detect specific T cells in a blood donor with an MHC class II background predicted to respond to a single spike-derived 15-mer. At first glance, it might be surprising that we identified reactive cells in both the CD3^–^CD4^+^ and CD3^+^CD4^+^ compartments. It needs to be considered, however, that a given peptide might induce polyclonal responses as has been demonstrated for influenza (39), resulting in clonotypes with different functional avidity, that may or may not downregulate CD3 upon stimulation.

Focusing on patients with preexisting conditions, we found individuals under immunosuppressive therapy, but not under hemodialysis, to harbor significantly reduced frequencies of specific CD3^–^ T cells as compared with age-matched controls (Figure 7). It is tempting to speculate whether this phenomenon could be simply explained by direct therapy-dependent suppression of vaccine-specific T cell activation, expansion, and memory differentiation or might involve a recently described mechanism where recurring herpesvirus infections, being common in immunosuppressed individuals (40), lead to contraction of the naive T cell pool in aged individuals, thereby limiting vaccination success (41).

Another important aspect of our study is the fact that only frequencies of CD3^–^ but not of conventional CD3^+^ T cells correlate with SARS-CoV-2 S1 domain–specific antibody titers, suggesting a more productive interaction of the latter with B cells. It needs to be determined whether this involves augmented cytokines or relies on other features regulating lymph node crosstalk during priming and/or memory formation.

Limitations of the data presented herein include the absence of an individual follow-up of spike-specific CD3^–^ T cells over time that is hampered by different SARS-CoV-2 infection and revaccination fates within our cohort; we were only able to provide information on spike-specific T cell stability in a cross-sectional manner. Therefore, it remains to be addressed how the specific CD3^–^ pool is quantitatively, functionally, and metabolically maintained in the absence of antigenic triggers. Our findings on CEF responses suggest that high-avidity CD3^–^ Th cells are kept over decades when recruited early in life, since CMV infection prevalence already reaches up to 36% in healthy individuals aged 6–11 years (42). It cannot be ruled out, however, that maintenance of high-avidity cells is dependent on recurring viral reactivation and might be critical to maintain functional superiority. In that context, data from animal models indicate that functional avidity might not be static but could undergo dynamic changes depending on antigen dose (38). For a comprehensive characterization of high-avidity CD3^–^CD4^+^ T cells, follow-up studies should include transcriptome and TCR repertoire analyses, allowing a more profound comparison with their CD3^+^CD4^+^ counterparts. Finally, although the mechanistic steps leading to downregulation of components of the TCR/CD3 complex have been examined in greater detail, including dynamics of internalization, degradation, and prevention of recycling (43–45), the exact physiological relevance of such a process beyond limiting excessive inflammation (46) will still require future investigations. The same holds true for investigation of the exact impact of the CD3^–^CD4^+^ subset on antiviral protection that needs to be addressed in larger cohorts in a prospective manner.

In summary, our data suggest CD3^–^CD4^+^ Th cells as correlates of high-quality immune responses that may be used for general assessment of immunocompetence in vaccination-, infection-, or tumor-related immunity.

## Methods

### Study design and determination of humoral immunity.

Demographics of healthy probands are depicted in Table 1. Information on patients with comorbidities and age-matched healthy probands is summarized in Table 2. All participants were enrolled between January and April 2020 and received a standard 2-dose (21 days apart) SARS-CoV-2 mRNA vaccine (BioNTech/Pfizer BNT162b2 Comirnaty). Blood samples were collected on day 8 ± 1 after the second dose. All individuals were SARS-CoV-2 naive at the time of analysis, by frequent point-of-care testing and/or a negative SARS-CoV-2 nucleoprotein-specific ELISA (Euroimmun). Vaccination-induced SARS-CoV-2 spike S1 domain–specific IgG levels were analyzed in serum samples at day 8 ± 1 after the second dose by ELISA (Euroimmun). Samples were considered positive with OD ratios of ≥1.1 as per manufacturer′s guidelines. Samples exceeding the upper assay limit (OD ratio > 10) were prediluted and remeasured.

### Antigens.

Antigen-specific stimulations were conducted with an overlapping peptide mix consisting of 15-mers with 11 aa overlap covering the full sequence of the SARS-CoV-2 spike glycoprotein (Alpha variant, “Pepmix,” JPT). In one experiment, a single spike-derived 15-mer encompassing aa 816–830 was used (aa sequence: SFIEDLLFNKVTLAD; JPT). A combination of 15-mer peptide pools including CMV (“Peptivator pp65,” Miltenyi Biotec), EBV (“Peptivator consensus,” Miltenyi Biotec), and influenza H1N1 (“Peptivator matrix protein 1,” and “Peptivator nucleoprotein,” Miltenyi Biotec) were used for assessment of recall antigen–specific responses (termed CEF). Antigens were used at a concentration of 0.5 μg/mL per peptide unless otherwise indicated.

### Sample processing and stimulation.

Serum was collected and cryopreserved. PBMCs were isolated from heparinized blood by Ficoll-Paque density gradient centrifugation and immediately cryopreserved in liquid nitrogen. For analysis of antigen-specific T cells, 3 × 10^6^ to 5 × 10^6^ PBMCs per stimulation were thawed, washed twice in prewarmed RPMI1640 medium (containing 0.3 mg/mL glutamine, 100 U/mL penicillin, 0.1 mg/mL streptomycin, 20% FCS, and 25 U/mL Benzonase from Santa Cruz Biotechnology), rested for 2 hours in medium (RPMI1640 with glutamine, antibiotics, and 10% human AB serum, all Biochrom), and stimulated with SARS-CoV-2 spike or CEF peptide mix for 16 hours. Brefeldin A (10 μg/mL, MilliporeSigma) was added after 2 hours. DMSO was added to the unstimulated controls in the same quantity as in stimulated samples.

### Conventional and metabolic flow cytometry.

For surface stainings, antibodies against CD3 (SK7, BioLegend), CD4 (SK3, Becton Dickinson), CD8 (SK1, Ebioscience), CD45RO (UCHL1, BioLegend), CD62L (DREG-56, BioLegend), and PD-1 (EH12.1, Becton Dickinson) were used. In some experiments, anti-CD28 (CD28.2, BioLegend), -CD16 (3G8, BD), -CD56 (NCAM, BioLegend), and -CD94 (DX22, BioLegend) were used.

Undesired cells were excluded via a “dump channel” that contained CD14^+^ monocytes (M5E2, BioLegend), CD19^+^ B cells (HIB19, BioLegend), and dead cells (fixable live/dead, BioLegend). Cells were fixed in FACS Lysing Solution (Becton Dickinson), followed by permeabilization with FACS Perm II Solution (Becton Dickinson). Cells were intracellularly stained with anti-CD154 (24-31, BioLegend), anti-CD137 (4B4-1, BioLegend), anti–TNF-α (MAb11, BioLegend), anti–IFN-γ (4SB3, eBioscience), anti–IL-2 (MQ1-17H12, BioLegend), anti-Ki67 (B56, Becton Dickinson), and anti–IL-4 (MP4-25D2, BioLegend). Samples were measured on a BD Fortessa X-20 cytometer.

For key metabolic protein detection, representing major metabolic pathways, in antigen-specific T cells, antibodies were chosen that have been described and validated earlier (14, 15), as summarized in Figure 3A. Antibodies (all Abcam) were coupled in-house (CPT1a [8F6AE9]; IDH2 [EPR7577]; G6PD [EPR20668]; HK [EPR20839]; LDH [EP1566Y]) using lightning-link (Abcam) or antibody labeling kits (Thermo Fisher Scientific) or purchased ready to use (GLUT1 [EPR3915]; ACAC [EPR4971]; ATP5A (EPR13030[B]) and underwent extensive titration in combination with PMT voltration to achieve optimal FACS device sensitivity. PBMCs were stimulated and surface-stained as described before, followed by fixation and permeabilization with transcription factor staining buffer set (eBioscience). All metabolic markers were then stained in perm buffer together with CD154, CD137, and IFN-γ. For metabolic assessment, all samples were measured on a BD Fortessa X-20 cytometer in 1 batch together with single-stain compensation controls, thereby excluding any interassay variability.

### Generation and restimulation of spike-specific CD3^+^ and CD3^–^CD4^+^ T cell lines.

For generation of short-term antigen-specific T cell lines, 10^7^ PBMCs were stimulated for 16 hours with spike peptide mix in the presence of anti-CD40 (1 μg/mL, HB14, Miltenyi Biotec) for retention of surface CD154 expression (47). Thereafter, cells were surface-stained with anti-CD154 PE (24-31, BioLegend) and magnetically pre-enriched using anti-PE nanobeads (BioLegend) over a MACS LS column (Miltenyi Biotec). Spike-specific DUMP^–^CD154^+^CD137^+^ CD3^–^CD4^+^ and CD3^+^CD4^+^ subsets were further sorted to >97% purity on a FACSAria Fusion cell sorter (Becton Dickinson). Cells were expanded for 10 days with a 100-fold excess of autologous Mitomycin C–treated (MilliporeSigma) PBMCs in the presence of IL-7 and IL-15 (10 ng/mL each, Miltenyi Biotec). Cells were restimulated in the presence of CD3-depleted autologous PBMCs (1:5 ratio) for 16 hours.

### FACS data analysis.

FACS data were analyzed with FlowJo 10 (Becton Dickinson). The gating strategy for identification of antigen-specific CD4^+^ T cells is depicted in Figure 1. A T cell response was considered positive when antigen-stimulated PBMCs contained at least 2-fold higher frequencies of CD154^+^CD137^+^CD4^+^ T cells (stimulation index of 2) as compared with the unstimulated control with at least 20 events. Coexpression of cytokines was analyzed with the Boolean gating function in FlowJo.

### Sex as a biological variable.

The study examined men and women, and sex-dimorphic effects are reported.

### Statistics.

Statistical analysis and composition of ELISA and FACS data–derived figures were performed using GraphPad Prism 8. Data distribution was assessed using the Kolmogorov-Smirnov test. Depending on normal distribution or not, 1-way ANOVA (with Holm-Šídák post hoc) or Kruskal-Wallis test (with Dunn post hoc) was chosen for multiple comparisons. For 2-group comparisons, 2-tailed *t* test/Wilcoxon test (depending on data distribution; for paired data sets) or 2-tailed *t* test/Mann-Whitney *U* test (depending on data distribution; for unpaired data sets) were used. The relationship between 2 variables was examined by simple linear regression analysis. For analysis of contingency tables, Fisher’s exact test was applied. In all tests, a value of *P* < 0.05 was considered significant.

### Study approval.

The study protocol was approved by the ethics committees of the Charité–Universitätsmedizin Berlin (EA4/188/20), Universitätsmedizin Greifswald (BB 019/21, Greifswald, Germany), and Sachsen-Anhalt (EA7/21, Sachsen-Anhalt, Germany) and carried out in compliance with its guidelines. All participants provided written informed consent in accordance with the Declaration of Helsinki.

### Data availability.

All cellular data needed to evaluate the conclusions in the paper are present in the paper or the supplement. Values for all data points in the figures can be found in the Supporting Data Values Excel file. Requests for materials should be directed to the corresponding author.

## Author contributions

AS designed the study, conducted experiments, acquired and analyzed data, and wrote the manuscript. SG conducted experiments, acquired and analyzed data, and wrote the manuscript. VP, LMLT, AH, EVS, and KJ conducted experiments and acquired data. SL analyzed data. SMG, CC, and KK supervised the study and wrote and/or revised the manuscript.

## Supplementary Material

Supplemental data

Supporting data values

## Figures and Tables

**Table 1:**
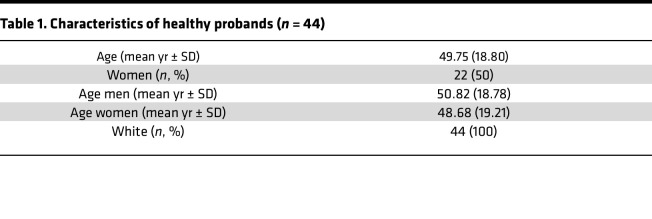
Characteristics of healthy probands (*n* = 44)

**Table 2:**
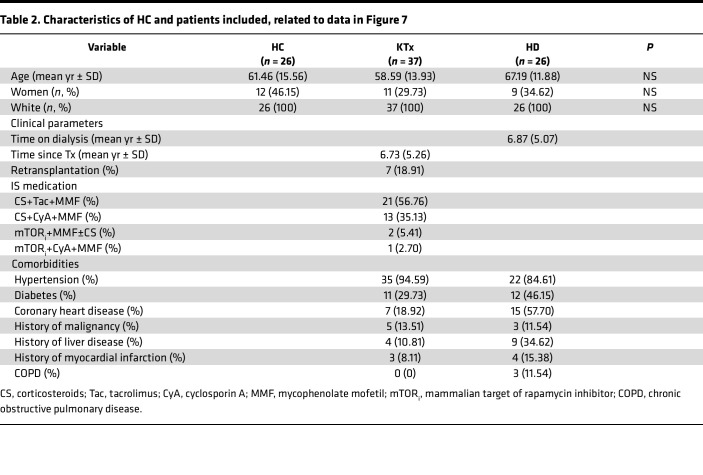
Characteristics of HC and patients included, related to data in Figure 7

## Version 1 01/11/2024

In-Press Preview

## Version 2 02/22/2024

Electronic publication
