# Supplementary material for: CD3 downregulation identifies high-avidity, multipotent SARS-CoV-2 vaccine– and recall antigen–specific Th cells with distinct metabolism
Source: JCI Insight. 2024 Jan 11;9(4):e166833. doi: 10.1172/jci.insight.166833 (PMC11143931; doi:10.1172/jci.insight.166833)
Supplement: Supplemental data [file jciinsight-9-166833-s010.pdf]

Supplemental Figure 1, Sattler et al.

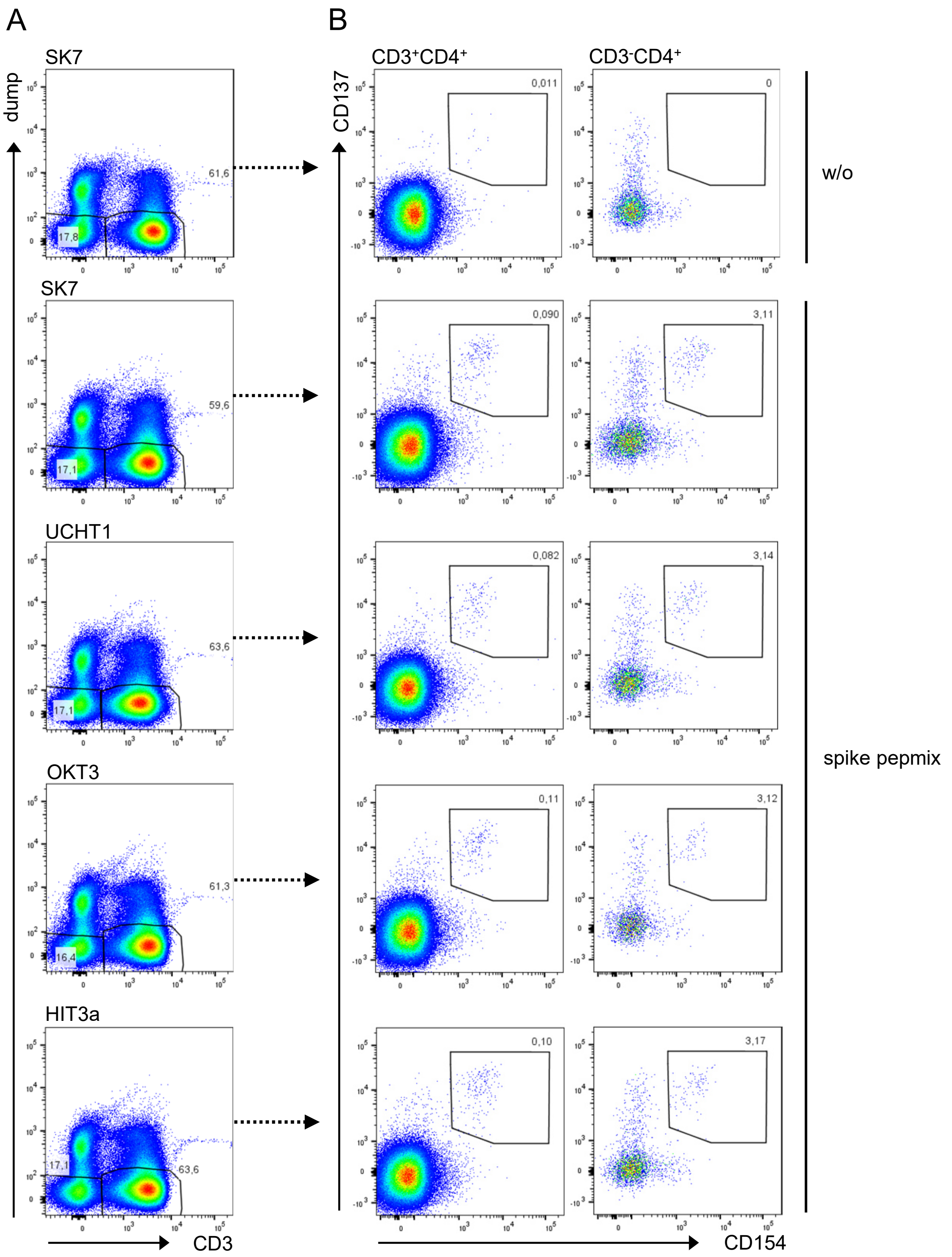

Identification of spike-specific $CD3^+CD4^+$ *and* $CD3^-CD4^+$ *populations with four different, commonly available anti-CD3 clones.* 10^7 PBMC were stimulated in one tube with spike pepmix. Thereafter, cells were split into four and separately stained with the complete surface panel, each including one of the depicted anti-CD3 PerCP-Cy5.5 conjugated clones (SK7, UCHT1, OKT3, HIT3a), respectively. Unstimulated cells served as control and were stained with a surface panel including anti-CD3 clone SK7. (A) Exemplary CD3 stainings for all four clones, showing similar brightness and population shapes, allowing consistent inter-sample gating. (B) After fixation, permeabilization and intracellular staining, spike-specific cells were identified after pre-gating on CD4 (not shown) as before according to CD154/CD137 co-expression. Plots show similar frequencies of specific T cells within the $CD3^+CD4^+$ and $CD3^-CD4^+$ populations, respectively, regardless of the anti-CD3 clone used. Data are representative for four different donors collected in two independent experiments.

Supplemental Figure 2, Sattler et al.

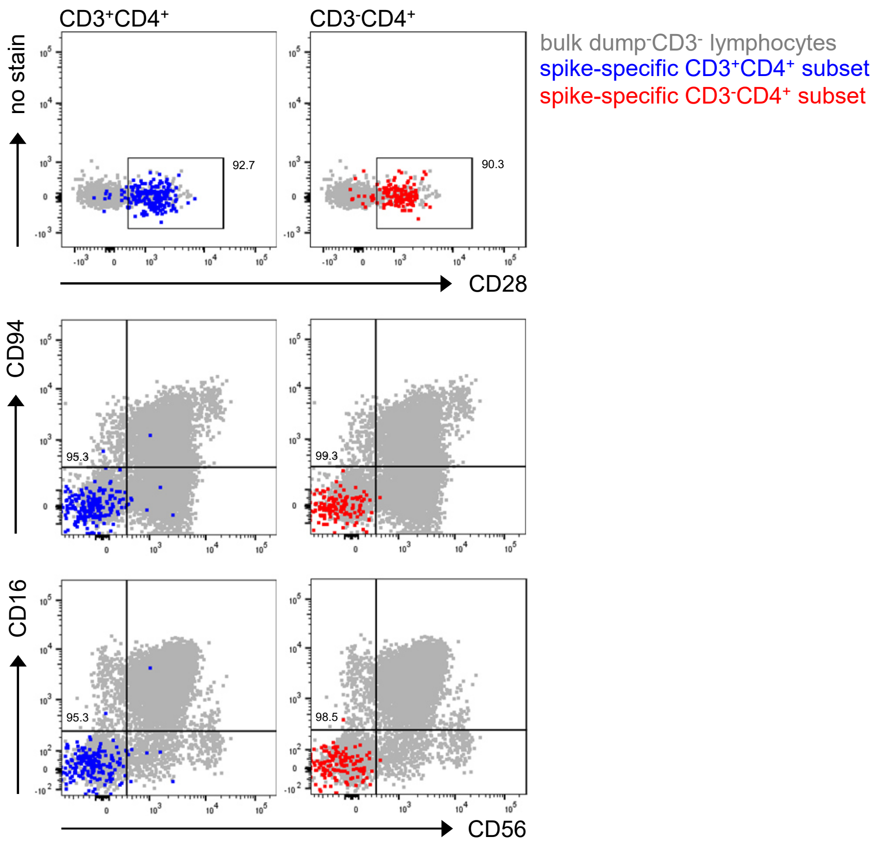

Spike-induced CD3$^-$CD4$^+$ cells are bona fide T cells. PBMC were stimulated with spike peptide mix, stained and pre-gated as before. Expression of the T cell co-stimulator CD28 or the NK cell associated markers CD56, CD94 and CD16 was analyzed in both spike-specific CD3$^+$CD4$^+$ (blue dots) and CD3$^-$CD4$^+$ (red dots) populations. In all experiments, the bulk CD3$^-$dump$^-$ lymphocyte population (grey) served as internal control. Specific cells were largely CD28$^+$ and devoid of NK cell markers, suggesting no contamination with non-T cells. Data are representative for 4 different donors collected in two independent experiments.

Supplemental Figure 3, Sattler et al.

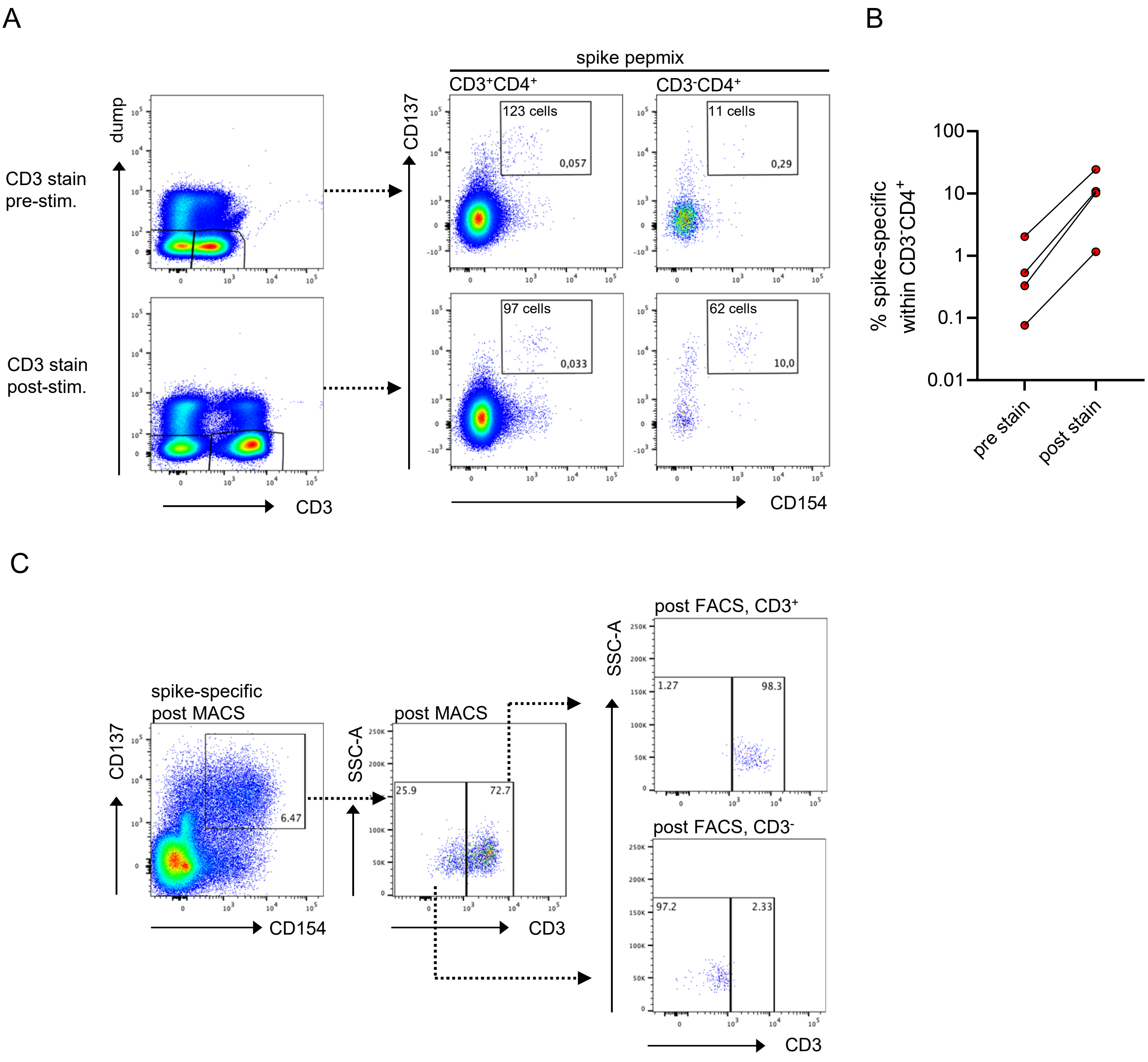

Stimulation-induced downregulation of CD3 in vaccine-specific Th cells. PBMC were surface stained for CD3 pre- or post-stimulation with spike pepmix as indicated. Frequencies (A and B) and counts (A) of vaccine-specific T cells were detected according to CD154 and CD137 co-expression within the $CD3^-CD4^+$ and $CD3^+CD4^+$ compartments. (C) Exemplary sorting strategy for spike-specific $CD3^-CD4^+$ and $CD3^+CD4^+$ Th cell subsets using MACS-pre-enrichment, followed by FACS purification, including post-FACS re-analysis.

Supplemental Figure 4, Sattler et al.

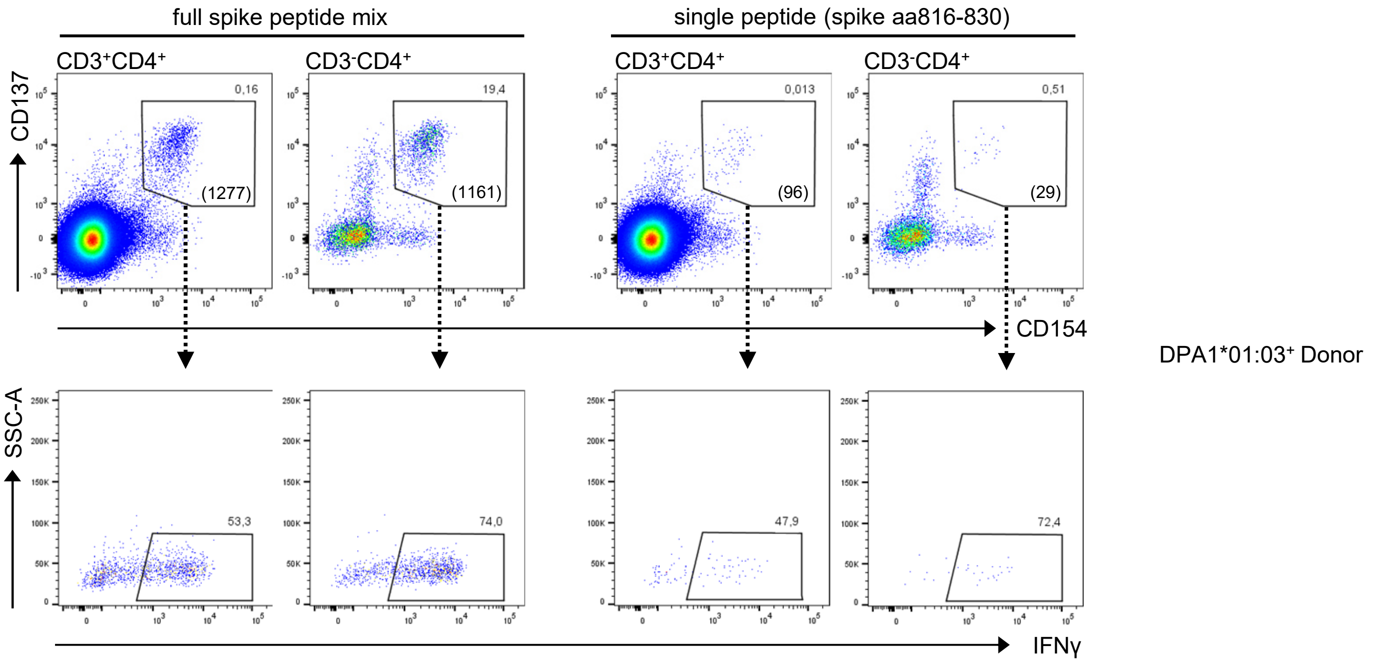

Spike derived single peptide response (aa816-830) in CD3⁺CD4⁺ and CD3⁻CD4⁺ subsets. PBMC of a HLA-DPA1*01:03⁺ Donor were stimulated with complete spike peptide mix or with a single spike-derived 15-mer (aa816-830) at 0.5 µg/ml per peptide as indicated. Frequencies and counts (in brackets) of specific CD154⁺CD137⁺ T cells were determined in both CD3⁺CD4⁺ and CD3⁻CD4⁺ subpopulations, along with quantification of IFN*γ* production.

Supplemental Figure 5, Sattler et al.

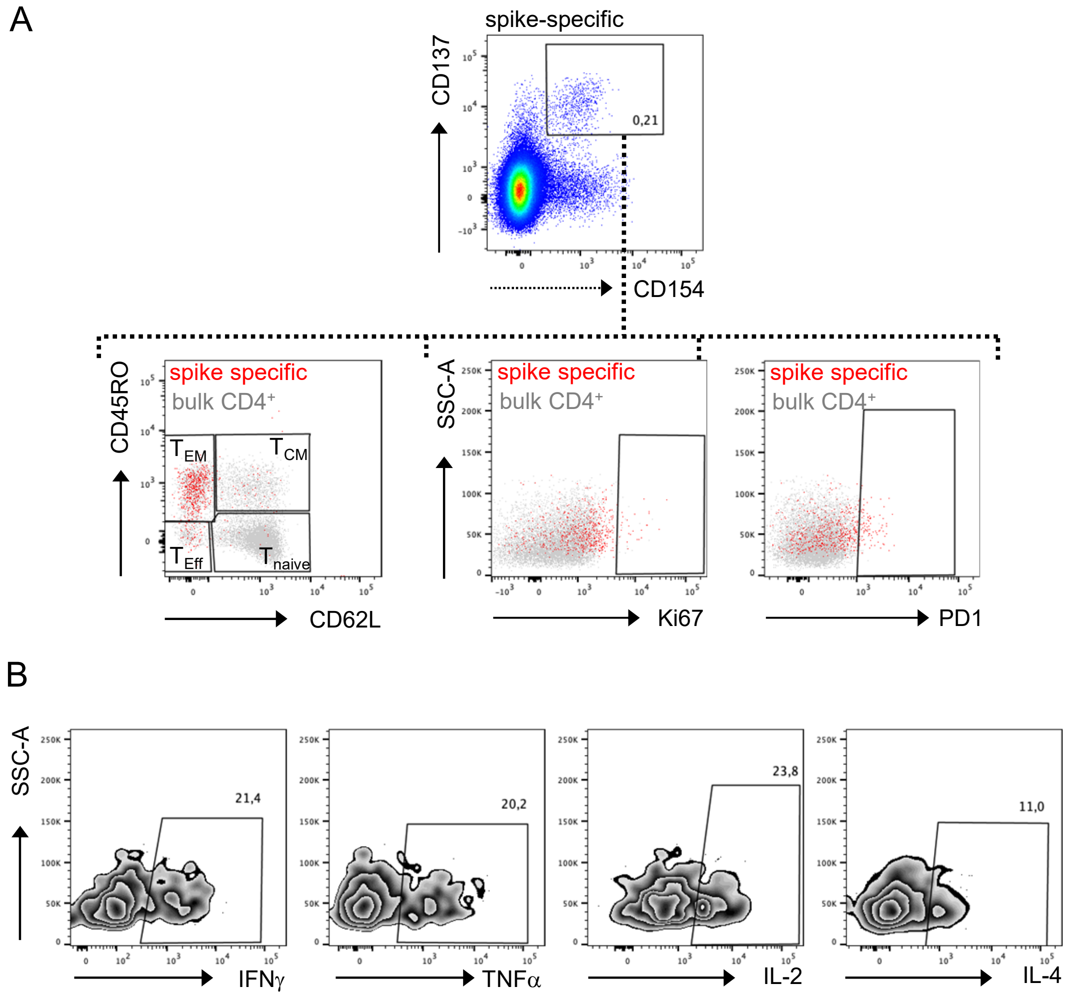

Phenotypic and functional assessment of antigen-reactive Th cell subsets. Exemplary gating strategy to quantify frequencies of (A) naïve/memory T cells according to CD45RO and CD62L expression (left; TEM - effector/memory-, TCM – central memory-, TEff – effector- and Tnaive – naïve Th cells), or Ki67 (middle) or PD1 (right) or (B) cytokine expressing Th cells within the antigen-specific $CD154^{+}CD137^{+}$ populations, equally accounting for both $CD3^{-}CD4^{+}$ and $CD3^{+}CD4^{+}$ Th cell subsets.

Supplemental Figure 6, Sattler et al.

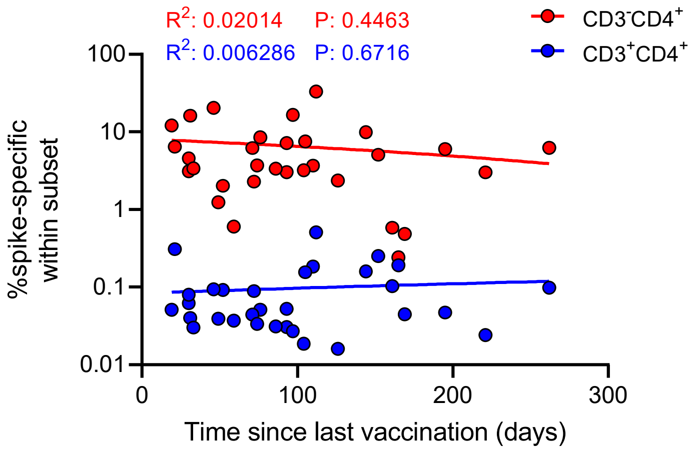

Correlation of spike-specific T cell frequencies with time since last vaccination. Spike-specific $CD3^+CD4^+$ and $CD3^-CD4^+$ T cells were identified as before in healthy virus-naïve probands in the follow-up period after SARS-CoV2 vaccination. Frequencies were plotted against days since last vaccine dose. Data derived from n=31 virus-naïve individuals. Statistical analyses were performed using simple linear regression.

Supplemental Figure 7, Sattler et al.

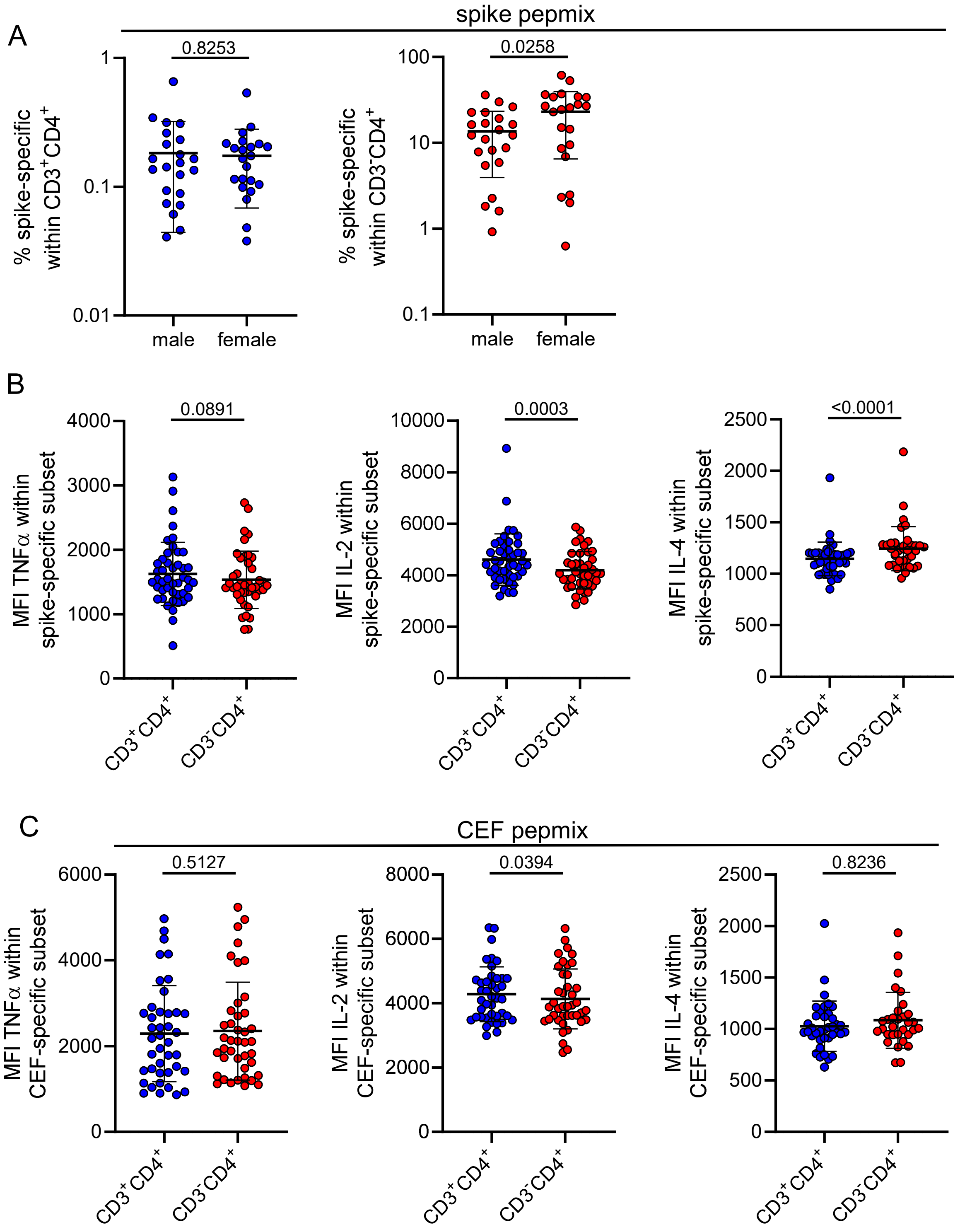

Sex distribution and per-cell cytokine expression levels of specific $CD3^-CD4^+$ and $CD3^+CD4^+$ Th cell subsets. Spike-specific Th cells were identified as before; frequencies of $CD3^-CD4^+$ and $CD3^+CD4^+$ subsets were quantified in probands stratified according to male (n=22) and female (n=22) sex. (B) Per-cell expression levels of TNFα, IL-2 and IL-4 according to MFI levels in spike-specific $CD3^-CD4^+$ and $CD3^+CD4^+$ Th cell subsets (n=42). (D) Per-cell expression levels of TNFα, IL-2 and IL-4 according to MFI levels in CEF-specific $CD3^-CD4^+$ and $CD3^+CD4^+$ Th cell subsets (n=41). Statistical analysis in (A) using unpaired Mann-Whitney-test and in (B) and (D) using paired, two-tailed Wilcoxon test. Where applicable, graphs show means ± SD.
